# 2396. In Nursing Home Residents, SARS-CoV-2 Boosters Reduced Sex Disparity in Vaccine Response Observed in Primary Series

**DOI:** 10.1093/ofid/ofad500.2016

**Published:** 2023-11-27

**Authors:** Brigid Wilson, Stefan Gravenstein, Sabra L Klein, Carson Smith, Anna Yin, Patrick Shea, Oladayo A Oyebanji, Christopher King, David Canaday

**Affiliations:** VA Northeast Ohio Healthcare System, Cleveland, Ohio; Brown University, Providence, RI; Johns Hopkins Bloomberg School of Public Health, Baltimore, MD; Case Western Reserve University, Cleveland, Ohio; Johns Hopkins, Baltimore, Maryland; Johns Hopkins, Baltimore, Maryland; Case Western Reserve University, Cleveland, Ohio; Case Western Reserve University, Cleveland, Ohio; VA Northeast Ohio Healthcare System, Cleveland, Ohio

## Abstract

**Background:**

In a study initiated in 2020, we recruited nursing home (NH) residents and collected blood samples serially after SARS-CoV-2 vaccinations. While sex differences in vaccine-induced immune response have been previously observed, similar vaccine efficacy across sex groups was reported for Moderna and Pfizer mRNA vaccines. Sex differences in immune response may diminish in very late life, so we sought to compare immune response to mRNA primary series and monovalent and bivalent boosters in NH residents, a frail elderly population.

**Methods:**

We analyzed anti-Spike antibodies and neutralizing titers to Wuhan strain for sex differences in immune response following primary series and subsequent booster doses. We summarized demographics and infection history of NH subjects with available sample data at each vaccine dose. Omicron BA.5 assays were compared for the second monovalent and bivalent boosters. Comparisons were stratified by prior infection at the time of sample. Samples collected about breakthrough infections were excluded. Geometric mean titers were calculated for each post-vaccine time and compared across sex groups using t-tests on log-transformed titers.

**Results:**

Despite new enrollment and loss to follow up, our cohort retained a sex balance ranging from 39% women, 61% men to an even split across four post-vaccine timepoints. Men and women had similarly advanced age and rates of prior COVID-19 over time (Table). Following the primary series, women with prior infection had significantly higher anti-Spike antibodies and higher neutralizing titers than men with prior infection (Figure 1). Following both monovalent booster doses and a bivalent booster dose, no sex differences were detected in these endpoints among those with prior infection. Neither were differences observed in infection naive subjects nor in titers for the Omicron BA.5 strain (Figure 2).

**Table 1:**
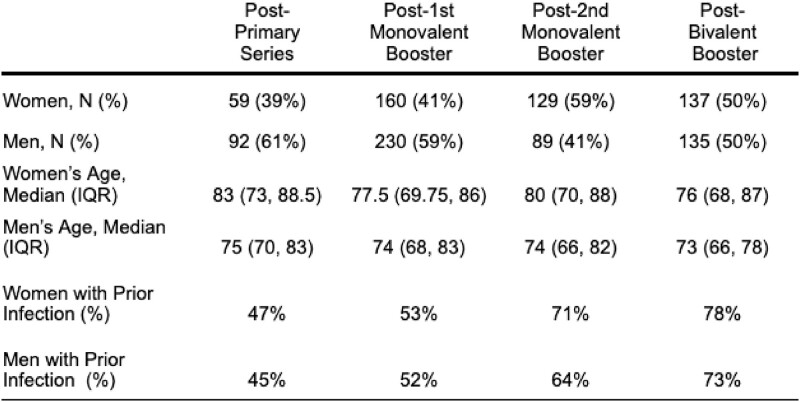
Demographics of NH residents with available samples by vaccine dose

**Figure 1:**
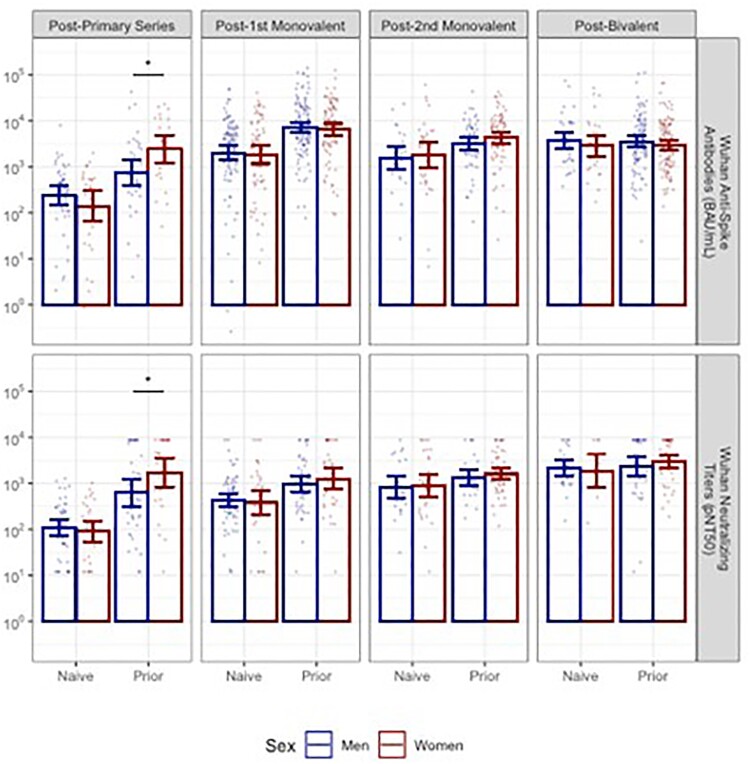
Post-vaccine Wuhan anti-Spike antibodies and neutralizing titers

Post-vaccine Wuhan anti-Spike antibodies and neutralizing titers by vaccine dose and sex, with bars showing geometric mean titers and 95% confidence intervals. Significant differences in GMT indicated with * at p < 0.05.

**Figure 2:**
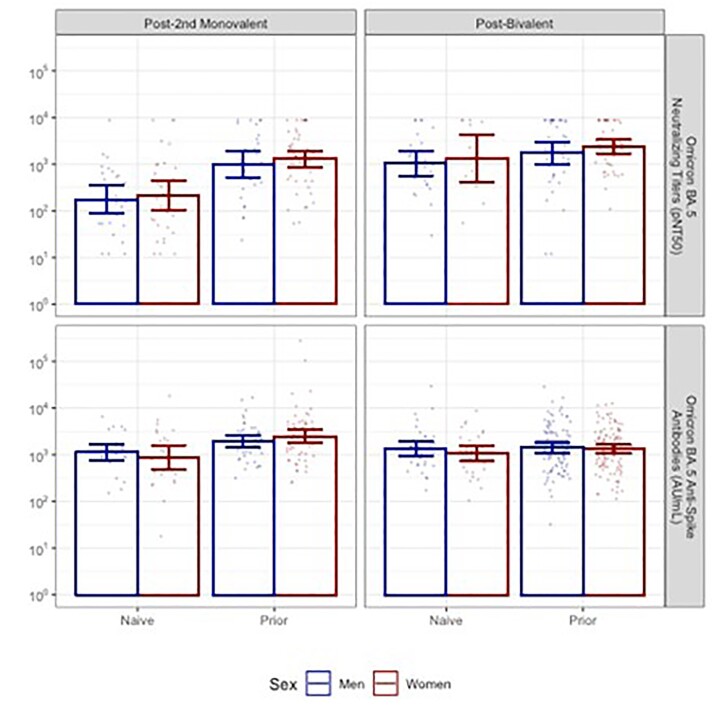
Post-vaccine Omicron BA.5 anti-Spike antibodies and neutralizing titers

Post-vaccine Omicron BA.5 anti-Spike antibodies and neutralizing titers by vaccine dose and sex, with bars showing geometric mean titers and 95% confidence intervals. No sex differences detected.

**Conclusion:**

Sex differences observed among NH residents with prior infection following primary series mRNA vaccination diminished with additional vaccine doses. Within NH residents, this may be a result of sex differences in attrition among the lowest responders. Further study is required to assess sex differences in the durability of immune response between doses and in T-cell response.

**Disclosures:**

**Stefan Gravenstein, MD, MPH**, CDC: Grant/Research Support|Genentech: Advisor/Consultant|Genentech: Grant/Research Support|GSK: Advisor/Consultant|GSK: Honoraria|Janssen: Advisor/Consultant|Janssen: Honoraria|NIH: Grant/Research Support|Pfizer: Grant/Research Support|Pfizer: Honoraria|Sanofi: Advisor/Consultant|Sanofi: Grant/Research Support|Sanofi: Honoraria|Seqirus: Grant/Research Support|Seqirus: Honoraria **David Canaday, MD**, Pfizer: Grant/Research Support

